# Viral Infections, Are They a Trigger and Risk Factor of Alzheimer’s Disease?

**DOI:** 10.3390/pathogens13030240

**Published:** 2024-03-08

**Authors:** Meagan D. Rippee-Brooks, Wenzhe Wu, Jianli Dong, Miguel Pappolla, Xiang Fang, Xiaoyong Bao

**Affiliations:** 1 Microbiology and Immunology Graduate Program, Department of Microbiology and Immunology, The University of Texas Medical Branch, Galveston, TX 77550, USA; 2 Department of Pediatrics, The University of Texas Medical Branch, Galveston, TX 77550, USA; 3 Department of Pathology, The University of Texas Medical Branch, Galveston, TX 77550, USA; 4 Department of Neurology and Mitchell Center for Neurodegenerative Diseases, The University of Texas Medical Branch, Galveston, TX 77550, USA; 5 The Institute of Translational Sciences, The University of Texas Medical Branch, Galveston, TX 77550, USA; 6 The Institute for Human Infections and Immunity, The University of Texas Medical Branch, Galveston, TX 77550, USA

**Keywords:** Alzheimer’s Disease, AD pathology, virus–AD hypothesis, Zika, herpesvirus, SARS-CoV-2, influenza

## Abstract

Alzheimer’s Disease (AD), a progressive and debilitating condition, is reported to be the most common type of dementia, with at least 55 million people believed to be currently affected. Many causation hypotheses of AD exist, yet the intriguing link between viral infection and its possible contribution to the known etiology of AD has become an attractive focal point of research for the field and a challenging study task. In this review, we will explore the historical perspective and milestones that led the field to investigate the viral connection to AD. Specifically, several viruses such as Herpes Simplex Virus 1 (HSV-1), Zika virus (ZIKV), and severe cute respiratory syndrome coronavirus 2 (SARS-CoV-2), along with several others mentioned, include the various viruses presently considered within the field. We delve into the strong evidence implicating these viruses in the development of AD such as the lytic replication and axonal transport of HSV-1, the various mechanisms of ZIKV neurotropism through the human protein Musashi-1 (MSI1), and the spread of SARS-CoV-2 through the transfer of the virus through the BBB endothelial cells to glial cells and then to neurons via transsynaptic transfer. We will also explore beyond these mere associations by carefully analyzing the potential mechanisms by which these viruses may contribute to AD pathology. This includes but is not limited to direct neuronal infections, the dysregulation of immune responses, and the impact on protein processing (Aβ42 and hyperphosphorylated tau). Controversies and challenges of the virus–AD relationship emerge as we tease out these potential mechanisms. Looking forward, we emphasize future directions, such as distinct questions and proposed experimentations to explore, that the field should take to tackle the remaining unanswered questions and the glaring research gaps that persist. Overall, this review aims to provide a comprehensive survey of the past, present, and future of the potential link between viral infections and their association with AD development while encouraging further discussion.

## 1. Introduction

Alzheimer’s Disease (AD) has challenged researchers for decades to attempt to uncover its elusive origins, multifaceted pathogenesis, and complicated causation. As we continue this quest for answers to ease the lives of aging people all over the globe, a compelling question arises and continues to resurface: could viral infections play a crucial role in the complex development of AD? As first described by Alois Alzheimer over a century ago, AD remains a pervasive health challenge, affecting millions of people worldwide [1]. AD is characterized by progressive, irreversible cognitive decline, memory loss, and the presence of classical neuropathology, often including: amyloid-beta (Aβ) plaques and neurofibrillary tangles [2], neuropil threads, and dystrophic neurites accompanied by astrogliosis and microglial cell activation, resulting in neuroinflammation [3]. We will consider historical and contemporary research findings that specifically link virus infections to their potential to induce and shape the progress of AD pathology. We will evaluate three specific viral contributions to AD here, not merely as speculative endeavors of contemporary research findings that specifically link virus infections to their potential in shaping the progress of AD pathology, but as a scientific inquiry supported by continued emerging evidence.

## 2. Overview of AD Pathology

To provide a solid foundation for exploring viral links to the development of AD, a comprehensive understanding of the pathological features of the disease is imperative. AD manifests as a progressive deterioration of cognitive functions, primarily encompassing the mutual characteristics of memory loss, impaired reasoning, and eventual debilitating disruptions to basic, everyday life [4]. Two primary cardinal lesions often associated with AD are amyloid-beta (Aβ) plaques and neurofibrillary tangles (NFTs) containing hyperphosphorylated tau (hyper-p-tau), although the presence of Aβ and Tau cannot completely conclude AD, as there are cognitively unimpaired individuals who can have biomarker evidence of both Aβ and Tau pathology but will often not develop clinical manifestations in their lifetime [5]. Other lesions include cerebral amyloid angiopathy, glial responses, and neuronal and synaptic loss [3]. Aβ plaques are composed of aggregated Aβ protein fragments that accumulate between nerve cells within the brain [6]. These extracellular proteinaceous deposits are implicated in disrupting neuronal communication and triggering inflammatory responses, which accelerate the progression of the disease [7,8]. NFTs result from the abnormal aggregation of tau protein within neurons due to hyperphosphorylation of tau. Neuronal function is thereby impaired in these patients affected with AD because their neurons are structurally unstable due to these intracellular tangles [9,10]. While genetics and aging are acknowledged and accepted contributors to the development of AD, emerging evidence suggests that infectious agents, specifically viruses, could influence or modulate the balance of molecular events leading to AD pathology [11,12]. There are studies that have highlighted the complexity in triggering or exacerbating AD including but not limited to genetic susceptibility, environmental influences, and the potential role of infections. Specifically, some of these studies highlight genetic susceptibility through well-described mutations in amyloid precursor protein (APP), presenilin 1 (PSEN1), presenilin 2 (PSEN2), and apolipoprotein E (APOE) genes, which account for only 30–50% of the heritability of AD [13]. Other studies have investigated the link of environmental risk factors or exposures such as traumatic brain injury (TBI), blood pressure, smoking, education, socioeconomic status, air pollution or geographical location, diet, and physical activity congruently with those previously mentioned gene interactions across individual’s age and duration of specific exposures [14,15]. Further studies have contributed to exploring the potential role of viral infections in AD either through direct methods of infection, and thereby modulation of neuronal function leading to neuronal damage, chronic neuroinflammation due to direct infection, processing of Aβ, tau protein dysregulation, and finally immune system dysregulation, or indirectly, through virus-induced inflammation and subsequently encephalitis. Considering the dynamic nature of AD pathology is not only crucial for elucidating its origins but is also vital for the development of robust targeted therapeutic interventions.

## 3. Historical Perspectives on Viral Links to AD

Early observations have hinted at a potential connection between viral infections and AD development. Surveying these historical perspectives reveals the gradual evolution of thoughts that led to recent considerations and investigations of this viral hypothesis in AD. During the mid-20th century, there were records by clinicians and researchers reporting peculiar associations between infectious events and cognitive decline generally [16,17,18,19,20,21]. Specifically, the persuasive work by Oskar Fischer in 1907 established AD pathology, and it was not until decades later, specifically during the 1980s, that infectious agents came under scrutiny for their potential influence on neurodegeneration. Attention slowly began to shift toward specific viral agents and their probable role in AD. The discovery of HSV DNA in postmortem AD brains really ignited the interest in the viral hypothesis of AD-contributing causation [22,23]. Subsequently, the presence of other herpesviruses, specifically human herpesviruses 6,7 (HHV-6,-7), Epstein–Barr virus (EBV), and cytomegalovirus (CMV), were surveyed in AD brains, which provided additional support to an already convoluted hypothesis [24]. As research progressed, questions regarding the nature of the association between viral infection and AD continued to emerge and grew more difficult to approach. For example, the concept of latent viral infections, particularly with individuals living with herpesviruses, gained prominence. Additionally, studies visited the possibility of viral reactivation within the aging brain, thereby highlighting the dynamic interplay between viruses and the neurodegenerative processes understood in AD. For example, HSV-1 (which will be elaborated on more throughout this review) has genetic factors, such as the apolipoprotein E isoform 4, APOE ε4, that assist HSV-1 invasion and latency establishment in the brain of APOE ε4 knockout mice [25]. HSV-1 also participates in the induction of Aβ and tau pathogenesis through virion particles encouraging the oligomerization and fibrilization of Aβ through physical interaction with the virus’ surface glycoprotein B along with multiple tau phosphorylation sites identified with HSV-1 infection in neuronal cells [26,27,28]. These approaches to this viral hypothesis, coupled with advancements in molecular and cellular biology, have encouraged researchers to probe deeper into the possible precise mechanisms underlying the viral hypothesis in AD. In the following sections, we will cover several important viruses and their potential contribution to AD.

## 4. Elaboration of Selected Viral Examples Implicated in AD and Their Respective Roles 

**Figure 1:**
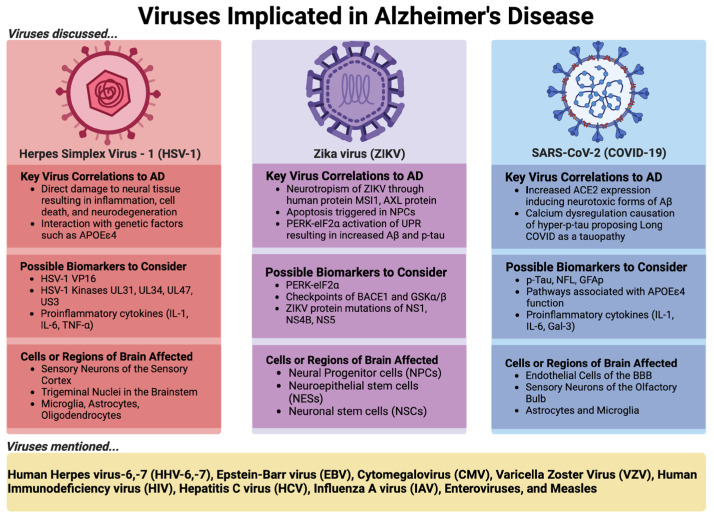
Virus examples implicated in AD. The specific viruses HSV-1, ZIKV, and SARS-CoV-2 are discussed here. A summary of the key virus correlations to AD, careful examination of the possible biomarkers to consider, along with cells or regions of the brain affected during infection that could be important to AD causation are highlighted. Figure created in BioRender.com accessed on 1 March 2024.

In this review, we use HSV-1, which has a long history of research on its contribution to AD, and two emerging viruses, ZIKV and SARS-CoV-2, as examples to discuss the virus–AD hypothesis in detail, as shown in Figure 1 below.

### 4.1. Herpes Simplex Virus 1 (HSV-1)

The original and most famous example of viral involvement in AD is Herpes Simplex Virus 1 (HSV-1). HSV-1 is a ubiquitous neurotropic double-stranded DNA viral pathogen that primarily infects epithelial cells of the oral and nasal mucosal regions. HSV-1 is a ubiquitous virus that affects more than 80% of people over 65 across the globe [29]. There exists a detailed and eloquent review by Shu Feng and colleagues that really delves into the details surrounding HSV-1 in AD [30]. We decided to add to the discussion, mainly in our future directions discussion, Section 4.1.4., below.

#### 4.1.1. Supportive Evidence

HSV-1 was first considered part of the microbe hypothesis in AD, which surfaced back in 1982, through the observation of individuals surviving a condition known as Herpes Simplex Encephalitis (HSE) and showing clinical signs reminiscent of AD in the forms of memory loss and cognitive impairment. To supplement this finding, brain regions affected in HSE (limbic system, and frontal and temporal cortices) were the same regions involved in AD [31]. Since Dr. McLauhlan’s 1980 work confirming HSV-1 presence in AD brains through in situ hybridization and Dr. Ball’s 1982 study linking HSV-1, HSE, and AD, there have been several research groups that have conducted investigations providing substantiative support of HSV-1 involvement in AD pathogenesis [32]. For example, the association between HSV-1 infection and AD shows mainly in people that harbor the APOE ε4 allele, and an antiviral agent acyclovir (ACV) was shown to amend AD-related tauopathy because this antiviral drug halts viral replication [33,34]. Cellular and viral kinases further exacerbate tauopathy and neurodegeneration which could be implicated by HSV-1 infection. GSK3β, induced by HSV-1 once inside the cell, and viral kinase US3 participate directly in phosphorylating tau to its hyperphosphorylated form and block apoptosis of the infected host cell, thereby driving latency reactivation events [35,36]. In addition, recurrent HSV-1 infection in a recently developed mice model induced hallmarks of neurodegeneration and cognitive deficits in mice [37]. Moreover, HSV-1-infected human-induced neural stem cells (hiNSCs) led to resembled changes observed in AD brains including amyloid plaque-like formation (PLFs), gliosis, neuroinflammation, and decreased functionality [38].

#### 4.1.2. Potential Mechanisms

HSV-1 has the propensity to enter sensory neurons near these mucosal regions through lytic replication and axonal transport, eventually reaching the trigeminal ganglion, where latent infection is established. Under stress from the host, usually in the form of a weakening immune system (which can be caused by diverse factors not elaborated on here), HSV-1 can undergo periodic reactivation cycles where the virus will travel back to the site of the primary infection through those same sensory neurons, usually causing clinical signs of lesions known as cold sores or oral blisters, but can also reach the brain by traveling from the bipolar trigeminal ganglion to the trigeminal nuclei in the brainstem and cause acute neurological disorders such as encephalitis, specifically HSE [39]. HSV-1 can also travel to neurons which project to the thalamus and reach the sensory cortex of the brain, causing HSE as well [39]. Entering the central nervous system (CNS) through the bloodstream is another possible neurological invasion mechanism of HSV-1 [40]. After entering the CNS, HSV-1 likely invades through receptors for HSV-1 (herparin sulfate proteoglycans), and intracellular damage, cell death, and neurodegeneration often result from direct neurological HSV-1 invasion through receptor-mediated entry [41]. Several receptors for HSV-1 are selectively enriched in the hippocampus of adult human brains, providing a plausible explanation for why this brain area is more afflicted in HSE patients [41]. Other than this possible mechanism linking HSV-1 to the development of AD through the virus directly damaging neural tissue [38], triggering an inflammatory response [42], or interacting with genetic and environmental factors to increase the risk of developing AD [43], there are also two other potential mechanisms of HSV-1. 

#### 4.1.3. Contrary Data

However, there are some instances not in support of this connection due to equivocal data. For example, two studies using small sample populations, <35 patients, reported no association between AD and levels of anti-HSV immunoglobulin G (IgG) [44,45]. Analysis of the data of the association between AD and the abundance of herpesvirus present in human brain samples needs to be carefully reassessed. Recently, a study supporting such a relationship in neurons was challenged [46]. The related claims pointed out several analysis errors, including mismatched gradients on color bars, which consequently cause the interpretation of p-values to look indistinguishable, and a lack of statistical robustness, subsequently establishing the study as statistically misleading [47]. Recently, HSV-1 has also been shown not to induce Aβ pathology in a mice model of late-onset AD [48].

#### 4.1.4. Possible Future Directions to Conclude the Role of HSV-1 in AD

Currently, some studies suggest a potential association between HSV-1 and AD, but the nature of this association remains unclear. There are several recent reviews that have listed and discussed many studies on the potential importance of HSV-1 in AD development [30,49,50,51]. Herein, we have dawn on some well-known or well-understood examples that we hope to continue to add to this perspective on HSV-1 and the infection process. This therefore allows us to find gaps to suggest possible experiments that will connect HSV-1 to AD development distinctly. The original work identifying the presence of HSV-1 in the brain of AD patients was confirmed by molecular detections, specifically through PCR and in situ hybridization [52]. In supporting the latter, in situ hybridization is used to identify the presence of virus proteins that could be present as the final products of viral particles. PCR techniques can detect viral DNA/RNA from inactive or degraded particles and are harmless to the host. Therefore, the association of HSV-1 infection with AD development by comparing the expression of viral DNA/RNA between the control group and AD groups is difficult to define by PCR experiments alone. Despite the high prevalence of herpesvirus infections, most of the time, our immune system can control it to be asymptomatic, while individuals with immune deficiency are linked to a higher risk of suffering from HSE [53]. Therefore, comparable HSV-1 immunoglobulin levels between control and AD groups cannot explain a role of HSV-1 in AD without considering comorbidity. Interestingly, AD itself seems to be a significant risk factor for HSV-1 infection, as APOE ε4 carriers are frequency linked to HSV-1 reactivation, supported by its presence in IgM-positive subjects or those with elevated levels of IgG, while no significant association was found in APOE ε4-negative subjects [54]. Herein, we will explore what has been found and what research could be further carried out to test the hypothesis of the contribution of HSV-1 infection to the development of AD. 

One important question to ask is whether the productive infection or the frequency of reactivation of HSV-1 is associated with AD onset or development. To address this question, comprehensive studies are needed. Since viral transcription, DNA replication, capsid assembly, and DNA encapsulations of HSV-1 occur exclusively in the nucleus [55], perhaps re-examining viral genome copies of nucleus extracts of commonly AD-affected areas in the brain such as the hippocampus, the frontal lobe, and the temporal lobe could be a way to investigate whether more genome copies, and subsequently more components for viral reactivation, are present in samples from AD-affected brains. Whether AD risk factors, including age, genetics, lifestyle, and environmental influence, affect HSV-1’s impact on AD should also be explored by subgroup and correlation analysis. Some viral proteins such as VP16 are shown to be important in lytic transcription [56]; therefore, quantifying viral proteins such as VP16 and comparing their abundance in healthy and AD groups could be helpful to determine the role of HSV-1 in AD. Statistics wise, a large sample size and more sophisticated analyses are needed for the correlation studies. 

Animal models are often instructive and insightful to comprehensively test the role of viral infections in AD onset and development. The capability of animal models in memory function assessment is also a surplus. Recently, it has been shown that recurrent HSV-1 by heat stress induces hallmarks of neurodegeneration and cognitive deficits in mice [37]. Researchers may use the model to further study whether anti-HSV-1 treatment blocks the disease consequences related to AD, including Aβ accumulation, tau hyperphosphorylation, neuroinflammation (astrogliosis and inflammatory cytokines/chemokines secretion), and/or cognitive deficits through measurable behavior testing. Whether HSV-1 mutants deficient in neuronal cell binding through glycoprotein B [57,58], HSV-1-encoded kinases that can phosphorylate tau to a hyperphosphorylated state [35], or nuclear egress of HSV-1 [59] lead to attenuated AD symptoms could also be explored. Other disease parameters to be visited are granulovacuolar degeneration [60], atrophy of the gyri in frontal and temporal cortices [61], and atrophy in posterior cortical areas [62] in functional imaging studies. These could also be carried out in animal models infected with HSV-1 or mutants. Taken together, HSV-1 as an etiological agent to AD development, like other viruses that we will discuss, and the viral hypothesis of AD remain a controversial and challenging field that needs further intense research mainly on the potential mechanisms of and consistent associations with specific viral species.

### 4.2. Zika Virus (ZIKV)

Zika virus (ZIKV), a neurotropic and neuroinvasive non-segmented, positive-sense single-stranded RNA arbovirus of the Flaviviridae family, has had a total of three global outbreaks over the last century, with the most famous epidemic taking place between 2015 and 2016 in the Americas and Europe [63]. ZIKV is mostly transmitted through the bite of an infected Aedes mosquito, but has also been horizontally transmitted through sexual activity, hospitalization, blood transfusion, or organ transplantation, and vertically transmitted from infected mother to fetus [64]. Usually, ZIKV is a self-limiting disease because most people with ZIKV do not have any symptoms, like most arboviruses. However, about twenty percent of people will experience ZIKV-induced inflammation including fever, rash, joint pain, and neurological impacts, such as rare reports where ZIKV has led to Guillain–Barré Syndrome (GBS) and microcephaly or ‘Congenital Zika Syndrome’ (CZS) [65,66,67,68,69,70]. Therefore, a link between AD pathology and ZIKV infection could exist, mainly because of the two etiological factors, inflammation, and neurological tropism with impact, that contribute to AD onset and development. 

#### 4.2.1. Supportive Evidence

One of the supportive pieces of evidence is the neurotropism of ZIKV. In neural progenitor cells (NPCs), neurotropism for ZIKV is canonical through the binding of the AUAG motif in the Xrn1-resistant RNA2 (xrRNA2) of ZIKV to human protein Musashi-1 (MSI1). There is also a non-canonical entry in NPCs through the interaction of the viral RNA structure AGAA tetraloop with human MSI1 [71]. Within human neuronal stem cells (NSCs), ZIKV also exhibits neurotropism due to the high expression of a cell surface receptor, the AXL protein, which is a receptor tyrosine kinase implicated in viral cell entry [72]. Because multipotent NPCs are targeted by ZIKV, embryogenesis is impeded due to the induction of apoptosis in these cells, which results in microcephaly through in vivo neonate models of ZIKV-infected mice [73]. Other than NPCs and NSCs, ZIKV has preference for infecting neuroepithelial stem (NES) cells and resulting in cell death, proliferation reduction, and a decrease in neuronal cell-layer volume [74]. ZIKV infection also causes structural disorganization and architectural impairment, which contributes to the observed deteriorating neurological defects of ZIKV [75,76]. 

Several groups have begun evaluating the possible connection between ZIKV and AD. A review published by Dr. Quincozes-Santos and colleagues covers thoroughly the role of glial cells in ZIKV-induced neurodegeneration. Here, they highlight cellular and molecular mechanisms of the inflammatory response, such oxidative stress, mitochondrial dysfunction, etc., in ZIKV-induced dysfunctional glial cells which can lead to the association with and progression of neurological complications, including those related to the aging brain [77]. A study by Drs. Kim and Kang explored the hypothesis that the persistent endoplasmic reticulum (ER) stress that ZIKV infection induces triggers the antiviral overactivation of the PERK-eIF2α pathway, and thereby results in synaptic failure, neuronal impairment, and cellular death. The PERK-eIF2α pathway also activates an unfolded protein response (UPR), resulting in increased presence of Aβ and phosphorylated tau (p-tau) through the upregulation of BACE-1 and GSKα/β [78]. Activated caspase 3, which is a cysteine protease activated in apoptosis, was suggested to be a factor in the functional decline of those affected by AD, and the infection of human mesenchymal stem cells with ZIKV leads to enhanced expression of caspase 3, thereby implicating that caspase 3 is involved in neuronal cell death and plaque formation in AD brains [79,80]. In one of the primary studies published over the last decade since the last epidemic of ZIKV, it has been shown that using an FDA-approved drug, known as memantine, which eases symptoms of AD, blocks N-Methyl-d-Aspartate receptors (NMDARs) through their overactivation and could disrupt the neuronal damage observed due to ZIKV infection. This study not only provides a unique use of NMDAR blockers but also confirms again that there exists a link between ZIKV and neurodegeneration, specifically in the form of AD [81]. 

#### 4.2.2. Contrary Evidence

However, of the reports of Aβ peptide acting as an antimicrobial agent through its putative protective function as a trap for pathogens [82,83,84], the group of Drs. Zhang and Zheng explored this possible connection of ZIKV with AD and showed that APP is stabilized by interacting with ZIKV and also inhibits ZIKV replication in both human neuronal progenitor stem cells and in neuronal stem cells in neonatal mouse brains. They found that the ZIKV-APP interaction prevents APP being cleaved via BACE-1 by blocking the BACE1 binding site for APP, resulting in a protective effect that reduces the availability of ZIKV to other cells. These data thereby implicate that APP is an antagonist for ZIKV through receptor mimicry [85]. We suggest that future research considers investigating processes upstream or downstream of Aβ peptide fibrilization and deposition which could affect its function as an innate immune protein that protects against pathogens, as previously mentioned. Do Aβ oligomers bind to ZIKV in the same way as it does with herpesvirus surface glycoproteins, thereby accelerating β-amyloid accumulation and possibly leading to a protective viral entrapment in vitro and in vivo [86]?

#### 4.2.3. Potential Mechanisms and Possible Future Directions to Conclude the Role of ZIKV in AD

ZIKV, compared with HSV-1, is a relatively newly considered virus in AD; therefore, there is not a clinical relevance study yet [87]. Although most ZIKV infections are characterized by subclinical or mild influenza-like illness, severe manifestations, especially those involved in neurological systems such as Guillain–Barré syndrome, also exist [87]. Therefore, following up with those patients and investigating the long impact of ZIKV on neurodegenerative diseases including AD will be instructive. Also, not all babies born from infected mothers have microcephaly [88]; therefore, following that population of babies to investigate the long impact of ZIKV on neurological development is also essential.

Despite one group suggesting that APP is an antagonist for ZIKV infection, the conclusion was made based on enhanced ZIKV viral RNAs in a medium of APP-null cells. The conclusion would be more concrete if both intracellular and extracellular infectious particles were quantified, as it is possible that intracellular APP entraps viral RNAs to facilitate the generation of infectious particles in cells, leading to reduced animal survival, while in APP-null cells viral RNAs are easily disseminated from cells or attacked by antiviral host factors which result in less infectious particles. Currently, animal models of ZIKV have demonstrated strong viral neurotropism enhanced by passive immunity with antibodies against other arboviruses. Different knockout models, such as Ifnar1^−/−^ [89] and Stat2^−/−^ [90], also have their effectiveness in recapitulating specific aspects of ZIKV pathogenesis and disease independently [91]. Perhaps researchers should consider memory decline through observing behavior and reporting AD-like features including changes in Aβ, p-tau, and/or neuroimaging after sham infection or infection with ZIKV using mouse models. ZIKV mutants which induce less neurological inflammatory responses could also be used to compare their impact on memory function with a wild-type (WT) virus. 

Other important experimental models for the ZIKV-AD hypothesis would be human-induced pluripotent stem cell (hiPSC)-derived three-dimensional (3D) cultures like brain organoids and spherical self-organized aggregates or hiPSC-derived neurological/immunoregulatory cells [92]. These models have been widely used for drug repurposing. Could repurposing established drugs lead to studies that investigate further the combination of other neuroprotective drugs used in AD to slow the impact that ZIKV could have on the potential development of AD? Or perhaps this could contribute to the drug development of an antiviral for ZIKV that also has preventative impacts on developing AD-like signaling if previous infections of ZIKV have occurred? Some other experiments that could be conducted to build upon these imperative studies are desperately needed to expand the field. For example, there are specific mutations that exist between the French Polynesian strain and the Brazilian strain of ZIKV in three of the non-structural (NS) proteins, three in NS1 (immune evasion), one in NS4B (evasion of type I IFN signaling), and one in NS5 (mask viral RNAs from viral RNA synthesis and replication) [93,94,95,96,97]. Designing mutant constructs and infection experiments in human neuronal progenitor or stem cell models and comparing their impact on the AD-like pathology or development with wild-type viruses should be completed. Brain organoid models could continue to be useful tools in studying how ZIKV is implicated in provoking AD pathologies. Experiments designed to repeat critical events after viral infection with ZIKV such as cytokine storm stimulation or blood–brain barrier (BBB) leakage with advanced organoids are needed to demystify the role of immune cells and blood vessels in potential mechanisms of neurodegeneration, mainly neuroinflammation. The development of complex brain organoid models could also be used to study the role of cell death and its contribution to cell populations involved, including but not limited to neurons, immune cells of the brain (mainly glia), and endothelial cells of blood vessels associated with the BBB. Perhaps neuroinflammation is not just due to the presence of ZIKV causing an infection, but also due to cell death or a lack thereof that the virus also triggers or blocks, which could be the reasoning for the acceleration in cognitive decline? Previous evidence shows that ZIKV likely plays a role in AD onset and/or development but we desperately need more studies to reach a decisive conclusion. Therefore, we hope this section promotes a rigorous discussion that encourages researchers to explore the proposed directions to ascertain definitively ZIKV’s role in the virus–AD hypothesis.

### 4.3. SARS-CoV-2 (COVID-19) or Long COVID

Recently, the entire world has been at the mercy of coronavirus disease 2019 (COVID-19), caused by the severe acute respiratory syndrome coronavirus 2 (SARS-CoV-2). SARS-CoV-2 is a positive-sense single-stranded RNA virus belonging to the family Coronaviridae [98]. Still today, there are thousands of new cases and deaths reported daily. While it is not as much of an urgent burden as it was just a couple of years ago, SARS-CoV-2 has made lasting impacts through its long-term effects or sequela from its acute or active infections, known as long COVID or post-COVID-19 conditions (PCC) [99]. Sadly, long COVID can include a wide range of ongoing health problems which can last in an individual for weeks, months, or even years. Anyone who has been infected with COVID-19 can experience this condition, which is variable between individuals, but there are some major common neurological symptomologies: headaches, difficulty thinking or concentrating (‘brain fog’), sleep problems, lightheadedness, changes in smell or taste, and depression or anxiety. A lot of these same symptoms are reported in AD. AD was found to be one of the most common COVID-19 comorbidities, and the virus infection contributed to an increase in mortality in these affected individuals. Of the two main pathways that have been proposed to explain how viruses are involved in the development of AD pathology, direct (microbes infect the brain and promote the accumulation of Aβ and hyper-p-tau) and indirect (inflammatory effects of an infection with microbes), it seems that SARS-CoV-2 could be implicated in both. There exists strong evidence that is suggestive of SARS-CoV-2’s ability to exhibit neurotropic properties, thereby allowing the virus to invade the CNS. Like the other sections of this review, we will take you through some of the viral lifecycle of SARS-CoV-2, paying particular attention to the entry of the virus, and we will mention how the virus could be contributing to the development of AD, but also any effective biomarkers that could lead to the implication of the viral causation of AD development. A review by Dr. Luisa Agnello’s group summarizes some of the most common critical biomarkers that may overlap between the two conditions [100]. 

#### 4.3.1. Supporting Evidence

The SARS-CoV-2 virus is transmitted through aerosolization of droplets riddled with the virus that are breathed in. In the upper respiratory tract, angiotensin-converting enzyme 2 (ACE2)-mediated entry plays a significant role in SARS-CoV-2 invasion [101]. Because of the case reports and meta-analysis that included data relating COVID-19 not only to the development of AD but also to other devastating neurodegenerative conditions such as Parkinson’s Disease (PD), amyotrophic lateral sclerosis (ALS), several other dementias, and multiple sclerosis (MS), there seems to exist a link between SARS-CoV-2 infection and neurodegenerative disease impact, especially AD [102]. Mechanistically, the BBB, consisting of endothelial cells, also expresses ACE2 receptors which can mediate the possibility of SARS-CoV-2 invasion into the CNS [103]. It has also been shown that viral infection from vascular endothelial cells through the BBB to the glial cells occurs, and then through infected neurons via transsynaptic transfer [104]. Usually, hematogenous spread through infected leukocytes, which operate as ‘trojan horses’ in this case, has also been suggested to carry the virus as leukocytes migrate to the brain [105]. Invasion of the CNS by the virus through the olfactory nerve to the olfactory bulb via retrograde axonal transport has also been described [104]. Within the brain, it has been reported that ACE2 can affect Aβ42 synthesis [91], and SARS-CoV-2 infection is also able to change the expression of ACE2 [106], suggesting the possibility of SARS-CoV-2 as an additional AD regulatory factor by controlling ACE2 expression and subsequently affecting neurotoxic forms of Aβ. 

After entering the CNS, several neurobiological outcomes caused by crosstalk between COVID-19 and AD have been also suggested by several groups. During SARS-CoV-2 infection, there are potential mechanisms that may be involved in the development of AD and its corresponding sequelae, which comprise Aβ accumulation, genetic factors like the pathway of the APOE ε4, neuroinflammation (signatures such as cytokines of IL-6, IL-1, and Gal-3), and microglial activation. Some common biomarkers that can be considered for neuronal injury during COVID-19 and AD include p-tau, neurofilament light chain protein (NFL), and glial fibrillary acidic protein (GFAp) microvascular injury [107,108,109,110,111,112]. These are all reported to be increased in both COVID-19 patients and AD patients [100,113]. Specifically, in regard to p-tau as a result of long COVID or COVID-19, we wanted to highlight the findings that increased calcium/cAMP/PKA and CaMKII activity, RyR (Ryanodine receptor) leakage, and dysregulated intracellular calcium levels in general are reported along with altered glutathione disulfide (GSSG)/glutathione (GSH), and importantly, this downstream has been implicated to be the causation of the observed hyper-p-tau in COVID-19 brains compared to controls. This thereby concludes that long COVID could be proposed as a tauopathy because of the previously listed reasons, along with the many residues which are phosphorylated such as S199, S202, S214, S626, and S356 [114]. Overall, SARS-CoV-2 can ignite AD-like signaling and, subsequently, post-COVID-19 neurological syndrome after CNS invasion [114,115,116].

The link between SARS-CoV-2 infection and AD is also supported by the observed impact of AD on SARS-CoV-2 infection. Patients with AD seem more vulnerable to SARS-CoV-2 infection, partially due to AD-induced direct and indirect pathological alterations, in addition to other AD-associated adverse impacts, including age, a lack of capabilities to follow recommendations on public health precautions, and nutritional factors [117,118,119]. Using a SARS-CoV-2 pseudovirus infection model, Dr. Shie’s group recently reported that the interaction between Aβ42 and SARS-CoV-2 strengthened the binding of the viral spike protein to ACE2, leading to more prominent viral entry, and subsequently enhanced levels of the inflammatory cytokine IL-6. These same outcomes were not observed with Aβ40 [120]. All of these reports support the link between SARS-CoV-2 infection and AD.

#### 4.3.2. Contrary Evidence

Because we still do not know much about how COVID-19 affects the body long-term, it is overall difficult to determine whether there is unsupportive or inconsistent evidence or whether the current evidence in support has absolute validity. However, based on an interaction study of the coronavirus S-protein binding with alpha-secretase (α-secretase), which functions as an integrin and metalloproteinase-9 (ADAM-9), it was suggested that S-α-secretase interaction produces a protective affect against AD through the adhesion of the virus to the cellular membrane and prevents the cleavage of the APP within the Aβ domain, thus preventing Aβ generation [121]. 

#### 4.3.3. Potential Mechanisms and Possible Future Directions to Conclude the Role of SARS-CoV-2 or long COVID in AD

A recent retrospective study utilized the electronic health records of at least 6 million American adults over the age of 65 that were infected or had a history of infection with SARS-CoV-2. From this study, it was determined through bidirectional relationships that these adults surveyed would have a significantly higher risk of developing AD [122,123]. While this study does address the growing concerns of viruses affecting individuals long-term, especially because of the signs and symptomology associated with coronavirus and brain function reported, these studies do not consider the prior health of the patients affected with COVID-19 and eliminate the possibility that these patients could already be demonstrating signs of AD prior to COVID-19 infection. Therefore, follow-up studies on the relationship between AD development and COVID-19 severity/symptomatic length of adult patients, with all comorbidities considered, will need to be carried out. Subgroup studies will also help to determine the impact of age, sex, race, and pre-existing health conditions on SARS-CoV-2’s effect on AD, if determined. 

Recently, a mouse model studying long COVID has been developed [124]. This model demonstrated that a mouse adapted to SARS-CoV-2 induces neuropathological outcomes several weeks after infection at similar rates of observed clinical prevalence of “Long COVID”. Establishing the viability of this model is a key step toward the rapid development of novel therapeutic strategies to ameliorate neuroinflammation and restore brain function in those suffering from the persistent cognitive dysfunction of “Long-COVID”. Using this model as a base, we could study the potential mechanisms that may be involved in the development of AD and its corresponding sequelae, which comprise Aβ accumulation, the expression of p-tau, NFL and GFAp, microvascular injury, genetic factors like the pathway of the APOE ε4, neuroinflammation (signatures such as cytokines of IL-6, IL-1, and Gal-3), and microglial activation [107,108,109,110,111,112]. Particularly, it could be noted that to further strengthen the knowledge of neuroinflammation during COVID-19 and its implication as a contributing factor to AD, experiments should investigate these cytokines and their effects in mouse models, such as with Tg2576 mice, which is a widely used mouse model for studying AD through the overexpression of human APP_695_ along with a Swedish mutation (KM670/671NL) under a hamster prion promoter that results in an elevation of Aβ levels and amyloid plaques [125], and APP^NL-F^ and APP^NL-G-F^ knock-in mice, both of which express the Swedish and Iberian mutations and accumulate Aβ and recapitulate amyloid plaques, synaptic loss, and neuroinflammation through astrocytosis and microgliosis [126,127]. Infecting those mice with SARS-CoV-2 to see if there is an acceleration and expression/production of AD-related biomarkers previously discussed or mentioned above could be a feasible and instructive study. 

The mechanisms obtained from mouse models can be further validated using primary or iPSC-derived neurological cells or iPSC-derived brain organoids. For example, once microglia-mediated inflammation is shown to play an essential role in AD onset and development in response to SARS-CoV-2 infection in mice, SARS-CoV-2-infected microglia could be used to study whether they can be polarized to a pro-inflammatory phenotype, leading to subsequent changes in neurodegenerative signaling. We could also compare the response of microglial cells to treatment with Aβ and infection. From there, inflammatory cytokines and chemokine secretion can be measured through expression and production.

## 5. Other Viruses Implicated in AD 

As shown in Figure 1 above, there are several other viruses, including human herpesvirus-6 (HHV-6), -7 (HHV-7) [43,128,129,130,131,132], Epstein–Barr virus (EBV) [133,134,135,136,137,138,139], cytomegalovirus (CMV) [33,140,141,142,143], varicella zoster virus (VZV) [144,145,146,147], human immunodeficiency virus [148,149,150,151,152,153,154,155,156,157,158,159,160,161,162], hepatitis C virus (HCV) [163], enterovirus [164,165,166], influenza A virus (IAV) [167,168,169,170,171], and measles [172,173,174,175], which have also been suggested to have a role in AD onset and development (see reference listed). Particularly, we wanted to briefly highlight the unique study by Levine et al. [176], where viral encephalitis and meningitis had the strongest risk association with an AD diagnosis, indicated through the mining of medical records of at least 300,000 individuals stored in FinnGen, a nationwide Finnish biobank. AD was at least 20 times more likely to be diagnosed in individuals who had experienced viral encephalitis compared to those who did not experience the condition. Strikingly, most of the viruses previously listed above are included in the 80% of viruses observed in Levine et al.’s study that can invade the nervous system and result in inflammatory responses. It must also be noted that the viral encephalitis diagnoses were described based on hospital billing codes, such as ICD-10 codes like A85 (which could be associated with enterovirus, adenovirus, and even arthropod-borne viral encephalitis [176]), but not on direct viral detection assays. Therefore, this begs for exploration of enterovirus encephalitis and its suggestive contribution to the development of AD (among other viruses listed above), especially since the majority of enteroviruses are strongly neurotropic and ubiquitous—with most individuals becoming infected with at least one of these viruses at some point in their life [166,177].

## 6. Summary of Potential Mechanisms on Viral Contribution to AD

**Figure 2:**
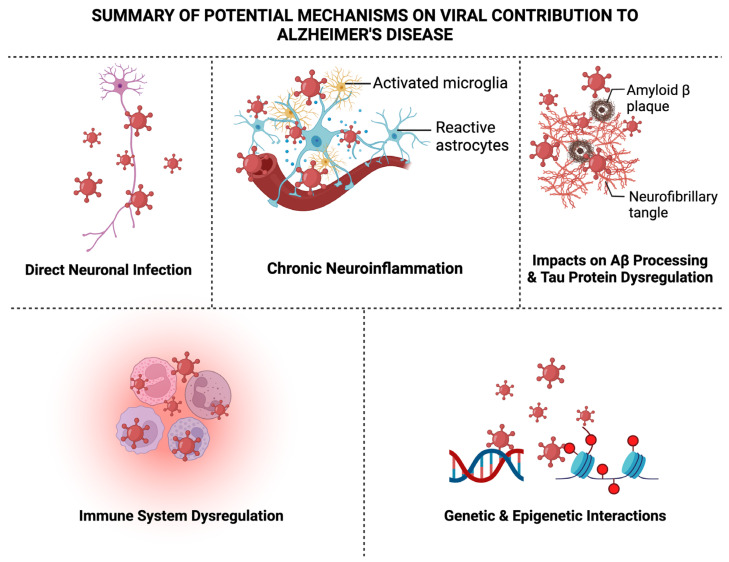
A summary of the potential mechanisms of viral contribution to AD. There are six acknowledged potential mechanisms of the virus–AD hypothesis, as illustrated above. These include the viruses discussed in this review through direct mechanisms such as directly infecting neurons, or through indirect mechanisms, such as chronic neuroinflammation resulting from activated microglia and reactive astrocytes, impacts on Aβ processing and tau protein dysregulation, immune system dysregulation, and lastly, genetic and epigenetic interactions that the virus induces on the host genome. Figure created in BioRender.com (accessed on 1 March 2024).

As discussed briefly throughout this review, there are several recognized potential mechanisms proposed for the virus–AD hypothesis which encompass direct neuronal infection, chronic neuroinflammation, altered Aβ processing, tau protein dysregulation, immune system dysregulation, blood–brain barrier disruption, and genetic and epigenetic interactions that may contribute to the development or exacerbation of AD, as shown in Figure 2, which we will briefly elaborate on next. 

### 6.1. Direct Neuronal Infection

Certain neurotropic viruses, such as various herpesviruses, establish latent infections within neuronal tissues. Their reactivation may result in direct neuronal infection, potentially contributing to AD pathogenesis due to the virus now replicating and synthesizing viral proteins, thereby causing whole virions to be produced, and causing acute infection. Because the virus can remain in the body and act as a lifelong reservoir despite its latency, this could suggest the involvement of these types of neurotropic viruses in AD, leading to synaptic dysfunction of neurons and subsequent neuronal death [178]. 

### 6.2. Chronic Neuroinflammation

Microglia, the resident immune cells within the brain, can be activated by viral infections and are an essential factor for AD pathogenesis. This activation can lead to the release of pro-inflammatory cytokines like IL-1β, TNF-a, and IL-6, contributing to chronic neuroinflammation observed in AD [179]. Consequently, the dysregulation of inflammatory signaling pathways has also been proposed because of viral infections. Viruses can frequently disrupt typical immune responses and perpetuate these pro-inflammatory environments conducive to AD development [180,181,182,183]. 

### 6.3. Impacts on Aβ Processing

Processing of APP may be influenced by viral infections, specifically by HSV-1. Altered APP processing could result in the accumulation of Aβ plaques, an etiological feature of AD pathology [184]. Interactions between specific viral components and Aβ have been proposed. This could be direct interaction between viruses and Aβ, which promotes aggregation and contributes to the seeding and spreading of plaque pathology in the brain [86]. 

### 6.4. Tau Protein Dysregulation

Chronic viral infections may influence the phosphorylation of tau protein. Viral-induced alterations in tau phosphorylation could contribute to NFT formation [185]. Just like with Aβ, tau can also interact, aggregate, and spread pathological tau due to chronic viral infections [186]. 

### 6.5. Immune System Dysregulation

Viral infections can dysregulate immune responses in the brain. Chronic viral infections compromise normal immune response, potentially contributing to the persistence of AD pathology [187]. Chronic viral infections may alter the function of immune cells responsible for clearing misfolded proteins. Impaired clearance mechanisms could exacerbate the accumulation of Aβ and tau in AD patients [188]. Viral infections could possess a potential role in disrupting the blood–brain barrier (BBB). This disruption may allow for the entry of peripheral immune cells and pathogens into the brain, contributing to neuroinflammation and AD pathology [189]. 

### 6.6. Genetic and Epigenetic Interactions

Genetic and epigenetic factors may influence susceptibility to viral infections and modulate the host response (e.g., immunity), thereby shaping the overall impact on AD development. For example, meta-analyses indicate associations between AD and viral infections with HSV-1, especially in individuals with the APOE ε4 allele [190]. With the development of RNA-seq and proteomics technologies, more common biomarkers for viral infections and neurogenerative diseases have been identified. These emerging biomarkers of interest include lipid biomarker 7-ketocholesterol. This pro-oxidant and pro-inflammatory molecule appears to significantly contribute to the development of AD and is produced during COVID-19 infections alike [191]. Non-coding RNAs (ncRNAs) are also another emerging regulatory family serving as study targets for the virus–AD hypothesis. For example, microRNA-146a-5p, thoroughly reviewed by Pogue and Lukiw, may be a special biomarker that can be used for both viral infection and inflammatory neurodegeneration and could be influenced by viral infections of the brain, such as SARS-CoV-2, and may subsequently lead to miRNA-regulated gene or protein changes conducive to AD development [192]. Recently, tRNA-derived RNA fragments (tRFs) have been shown to be essential regulators of many diseases including viral infectious diseases and neurodegenerative diseases [193,194,195,196,197,198]. Commonly impacted tRFs by both neurotropic viral infection and AD have also been identified. Whether there is tRF-mediated crosstalk between viral infections and AD development is also an interesting research topic for the research community. Iron metabolism could be an additional angle to test the virus–AD hypothesis. A retrospective study exploring the higher serum level of myoglobin as a predictor of the worse prognosis of COVID-19 infections was carried out recently [199]. In AD, there is an imbalance in iron homeostasis due to excessive iron contributing to Aβ accumulation and the formation of NFTs [200]. An evaluation of how iron metabolism during COVID-19 infection affects the development of AD has not been considered. The two conditions and their impact on iron metabolism and the outcome of disease have been studied separately, but exploring their roles together to identify a common pathogenesis pathway using myoglobin as a biomarker we hope could serve as a novel perspective. 

## 7. Challenges and Future Directions

Like any developing and evolving field, there will be challenges and controversies, and the virus–AD hypothesis is no different. Some of these key issues include causation challenges. Specifically, establishing causation is difficult, especially without longitudinal studies and intervention trials which could supplement the extensive use of observational studies. The need for methodological variability across studies poses a significant challenge. Differences in study design and sample sizes, overly complicated diagnostic criteria, and a lack of innovative detection methods for viral presence contribute to discrepancies in findings. Therefore, standardization is crucial to enhance reproducibility and reliability across virus–AD-linked studies. Animal models, while effective, also have limitations in recapitulating the complexity of human AD. These inconsistencies between animals and human responses to viral infections will continue to pose challenges in reasoning findings. We must continue to develop more accurate animal models to advance this standard of translational research. AD often coexists with various comorbidities, such as type 2 diabetes or even another viral infection such as HIV [201]. The combination of comorbidities is limitless, especially in human-based studies, thereby confounding the interpretation of virus–AD associations. These comorbidities must be identified and considered, as well as disregarded, so these findings can be compared. Future research should adopt a holistic approach by integrating, for example, genetic and environmental factors in AD development. This could lead to the refinement of risk prediction models and could enhance clinical approaches to the virus–AD hypothesis. Lastly, the sophisticated nature of the virus–AD hypothesis urgently needs collaborative efforts across various disciplines. Multidisciplinary approaches that involve virologists, neuroscientists, geneticists, and immunologists could unionize their diverse expertise and transform this field to achieve a more comprehensive understanding of the complexity of viral infections and AD. 

## 8. Conclusions

The virus–AD hypothesis explored here provides consistent associations between viral pathogens and AD pathology that cannot be ignored. While these key findings point to intriguing associations and potential mechanisms, the field continues to struggle with methodological challenges. There is still a great deal of research that needs to be conducted to establish a direct link between AD and viral infection. The journey ahead demands a commitment to rigorous research, innovative methods, and collaborative approaches. We must explore this viral hypothesis from all angles, answering the critical question of whether AD may merely predispose people to viral infections or vice versa. In our pursuit to understand, treat, and hopefully cure AD, as researchers, we must remain open, collaborative, and transformative, exploring where the science will lead us.
